# Factors Affecting Self-Care Behavior Levels among Elderly Patients with Type 2 Diabetes: A Quantile Regression Approach

**DOI:** 10.3390/medicina55070340

**Published:** 2019-07-04

**Authors:** Min Young Kim, Eun Ju Lee

**Affiliations:** 1 Department of Nursing, Ulsan University, Ulsan 44610, Korea; 2 College of Nursing, Keimyung University, Daegu 42601, Korea

**Keywords:** elderly, diabetes, self-care, quantitative methods

## Abstract

*Background and objectives:* Identifying factors that affect self-care according to low, middle, and high self-care levels among elderly patients with diabetes is the best way to prevent various life-threatening complications, and this can be accomplished by using an individualized approach to improve self-care. A quantile regression model is beneficial for estimating such factors because it allows the consideration of the entire conditional distribution of a dependent variable as it relates to independent variables. The objective of this study was to identify factors that affect self-care among elderly patients with diabetes using quantile regression. *Materials and Methods:* A cross-sectional survey of elderly patients with diabetes was conducted using the Self-Care Scale and six other related scales at three medical health centers in South Korea. *Results:* In the 10% quantile, the factors affecting self-care were age, smoking within the past six months, being educated about diabetes, depression, knowledge related to diabetes, self-efficacy, diabetes distress, and family support. Additional factors were as follows: age, smoking within the past six months, self-efficacy, and diabetes distress in the 25% quantile; age, self-efficacy, perceived health status, and diabetes distress in the 50% quantile; age, self-efficacy, perceived health status, and diabetes distress in the 75% quantile; and self-efficacy and perceived health status in the 90% quantile. *Conclusions:* Based on the results of this study, suggestions include providing education for sub-groups incapable of self-care, teaching stress management strategies, and increasing family support. In addition, for individuals capable of self-care, simplified programs that consist of self-efficacy improvement and stress control strategies are necessary.

## 1. Introduction

Diabetes is a worldwide disease that has increased the need for clinical and community health care efforts. The global prevalence rate of diabetes increased from 4.7% in 1980 to 8.5% in 2014 [1]. In the case of South Korea, the prevalence of diabetes increased from 6.1% in 2008 to 8.2% in 2017 [2]. For this reason, health authorities are putting great effort into health management initiatives for patients with diabetes, but the incidence of complications and the number of patients who are negligent in managing their diabetes, especially among the elderly, have increased [3].

Furthermore, patients diagnosed with diabetes must typically maintain self-care of the disease for the rest of their lives, as it is very difficult to recover completely from diabetes [4]. Elderly patients with diabetes have shown low self-care levels in managing their symptoms and have difficulty adhering to self-care behaviors for long periods of time [5,6]. The best way is to control blood glucose through proper self-care, which has been identified as crucial for keeping diabetes under control [4].

Previous studies have identified factors that influence decreased levels of diabetes self-care: male gender, low educational level, low economic status, long disease duration, drinking and smoking within the past six months, as well as religious or spiritual beliefs as general characteristics [6,7]. In addition, researchers found that self-care was negatively affected by low self-efficacy [6,8], lack of social support [7,9], distress and depression [8], severity of health status [10,11], and lack of knowledge about the disease [5,12]. Additionally, in previous studies, self-efficacy has been associated with better chronic disease self-care among individuals managing chronic diseases [8,13]. Self-efficacy, which is defined as the belief in one’s ability to achieve goals in specific situations [14], has been identified as a variable that should be addressed in self-care improvement programs for elderly patients with diabetes, as its influence on self-care is expected to be high [15,16]. In one study [8], self-efficacy, including social and family support, was described as a protective factor that enables patients with diabetes who suffer from depression and stress to perform effective self-care. The results of most previous studies indicate that in order to analyze the factors that affect self-care in this population, self-efficacy, as well as social and family support, should be included in the analysis [6,7,8,16]. Elderly people who experience chronic diseases often suffer from depression. 16]. When chronic disease is accompanied by depression, the risk and severity of disease-related complications tend to increase [17].

Therefore, to identify the factors that affect self-care among elderly patients with diabetes, the influence of all variables that have been identified as being correlated with or affecting self-care needs to be analyzed. However, previous studies have shown limitations in explaining factors that affect self-care, because while they all included general demographic characteristics, no study has included all the other variables in the analysis. Moreover, if the factors that affect the variable and mean self-care scores of elderly patients with diabetes are analyzed, the results will support the development of customized self-care programs and counseling for these individuals. In order to identify factors that affect self-care, a combination of statistical analysis methods needs to be applied.

Most previous studies involving elderly patients with diabetes investigated only the factors affecting mean self-care scores using the ordinary least squares (OLS) model and did not identify how the factors differ according to differing self-care scores. Sigurdardottir’s diabetes self-care model emphasized that the factors affecting self-care need to be examined according to low, middle, and high levels, because the level of self-care can be controlled by the individual patient [18]. For individual patients to be examined according to low, middle, and high self-care levels, a different statistical methodology is needed. It is true that the OLS model used for factor analysis in previous studies is effective for estimating the effects of independent variables on the average value of dependent variables [19]; however, OLS is limited in its ability to estimate the influence of independent or exogenous variables based on the mean scores of dependent variables [19]. In addition, when there is a possibility that certain characteristics are common in a group of people in which the distribution of variables is relatively low or high, OLS may either overestimate or underestimate the effects of certain variables [19]. In contrast, a quantile regression (QR) model is advantageous, as it analyzes the influence of individual independent variables based on the values of dependent variables, or the setting of each quantile, rather than the influence of the general average of dependent variables [19,20,21]. In order to analyze the factors that affect self-care according to self-care levels among elderly patients with diabetes, the use of a QR model is beneficial because the model estimates allow the consideration of the entire conditional distribution of a dependent variable as it relates to independent variables [19,20].

Therefore, this study employed QR to identify the effects of factors that affect self-care according to the levels of self-care among elderly patients with diabetes in S. Korea. The study findings will help nurses and other clinicians to better understand individual patients with diabetes and the factors that affect their self-care. Moreover, these findings can be used as a basis for developing strategies related to diabetes management programs in the healthcare system. The aim of the current study was to examine the levels of self-care related factors and investigate the factors that affect self-care according to the levels of self-care among elderly patients with diabetes using quantile regression.

## 2. Materials and Methods

### 2.1. Design

This study employed a cross-sectional survey to identify the factors that affect self-care according to self-care levels among patients with diabetes.

### 2.2. Sample and Setting

The study participants were elderly patients with diabetes who were registered with 1 public health center and 2 medical clinics in South Korea. We recruited study participants from this particular region because its elderly population has the highest rate of diabetes among the 8 administrative districts in the country [2]. The sample size was calculated using G Power 3 software. To have an effect size of 0.15 and a power of 0.8, the calculation indicated that at least 172 participants were required. Assuming an attrition rate of about 20%, we recruited a total of 200 participants. Under the inclusion criteria, participants had to be aged ≥65 years, to have been diagnosed with diabetes more than 1 year prior, and to be able to communicate with the researchers, and thus be without any diseases related to cognitive dysfunction (e.g., dementia or Alzheimer’s). The duration of the disease was set to at least 1 year. Finally, 198 participants were included in the study; 2 individuals were excluded because they were not able to properly respond to the survey. 

### 2.3. Data Collection

Researchers visited the directors of 1 public health center and 2 medical clinics in person and obtained their permission for data collection. The overall recruitment strategy at all 3 institutions included an informational session for the health care providers and the director of the medical clinics, and a display of informational materials such as posters and brochures in the waiting areas for patients. Patients who were identified as eligible and expressed an interest in the study first met with a researcher to confirm participation. If the researcher determined that the patient was eligible, the study’s objectives, procedures, and any additional information were explained. Following this, informed consent was obtained. 

The research assistants were 4 nurses who received 3 h of training concerning the questionnaire contents and the research procedure. All recruited study participants were directly approached and individually interviewed by the nurses.

### 2.4. Ethical Consideration

Ethical considerations for study participants were reviewed and approved by the university’s Institutional Review Board (IRB No. 1041386-20140515-HR-002-03/2014.05.15). In accordance with the ethical process, all participants were given information about the study’s objectives, procedures, and ethical considerations and then signed an informed consent form.

### 2.5. Instrument

#### 2.5.1. Self-Care Behavior

The Self-Care Behavior Scale, developed by Kim [22], was used to measure the self-care behavior of diabetes patients. The questionnaire includes 20 items with a 5-point Likert scale, with higher total scores indicating better self-care behavior. The reliability coefficient of the questionnaire was estimated as 0.84 in Kim’s study [22] and 0.92 in our study.

#### 2.5.2. Depression

The Geriatric Depression Scale Short Form (GDSSF) was developed by Yesavage and Sheikh [23] to measure the severity of depression in the elderly. This scale was translated and modified by Gi [24] to form the Korean version (GDSSF-K). The questionnaire consists of 15 items with a 2-point Likert scale. A higher total score indicates a more serious depression. The reliability coefficient of the GDSSF-K was estimated as 0.88 in Gi’s study [24] and 0.87 in our study.

#### 2.5.3. Diabetes Knowledge

A diabetes knowledge instrument was developed by Park [25] to identify how much patients with diabetes know about the disease. This instrument consists of 20 questions with a dichotomous scale. Total scores range from 0 to 20, and higher scores indicate that patients are more knowledgeable about diabetes management. The reliability coefficient of the questionnaire was estimated as 0.94 in Park’s study [25] and 0.75 in our study.

#### 2.5.4. Self-Efficacy

The Self-Efficacy Scale was developed by Hurley [26] and modified by Cho [27]. This questionnaire consists of 10 items with a 4-point Likert scale. Total scores range from 10 to 40, and higher scores indicate greater self-efficacy. The reliability coefficient of the questionnaire was estimated as 0.89 in Hurley’s study [26], 0.80 in Cho’s study [27], and 0.75 in our study.

#### 2.5.5. Perceived Health Status

To measure perceived health status, we used the Perceived Health Status Scale developed by Lawston [28] for diabetes patients. The questionnaire consists of 3 items with a 4-point Likert scale ranging from 1 (very bad) to 4 (very well), and higher total scores indicate better perceived health status. The reliability coefficient of the questionnaire was estimated as 0.76 in Lawston’s study [28] and 0.74 in our study.

#### 2.5.6. Diabetes Distress

The Diabetes Distress Scale was developed by Polonsky et al. [29] and translated by Choi [30] for Korean diabetes patients. The questionnaire includes 17 items with a 5-point Likert scale, and higher total scores indicate higher diabetes distress status. The reliability coefficient of the questionnaire was estimated as 0.87 in Choi’s study [30] and 0.72 in our study.

#### 2.5.7. Family Support

The Family Support Scale was developed by Cobb [31] and revised and translated by Gu [32]. This questionnaire consists of 12 items with a 4-point Likert scale. Higher total scores indicate better family support. The reliability coefficient of the questionnaire was estimated as 0.77 in Gu’s study [32] and 0.84 in our study.

### 2.6. Statistical Analysis

SPSS Statistics version 21.0 (SPSS, Chicago, IL, USA) and Stata version 12.0 were used to analyze the survey data. Using SPSS Statistics, the reliability coefficients of the instruments were estimated by calculating Cronbach’s alpha values. Analysis of variance and independent *t*-tests were used to examine differences in self-care scores with respect to diabetes-related characteristics of elderly patients with diabetes. In addition, employing SPSS Statistics, the survey data were analyzed using Pearson’s correlation to identify correlations between self-care scores and the other study variables. The Durbin–Watson score, the variance inflation factor (VIF), and multiple linear regression for OLS were applied to investigate the factors affecting self-care behavior. Finally, Stata version 12.0 was used for QR analysis to identify such factors according to levels of self-care among elderly patients with diabetes.

## 3. Results

With respect to the demographic and disease characteristics of the 198 study participants (elderly patients with diabetes), the highest self-care scores were attained by participants who were 65 to 69 years old (F = 7.76, *p* < 0.001), had a high-school education (F = 3.76, *p* = 0.012), had high economic status (F = 3.21, *p* = 0.003), and had not drunk alcohol within the last 6 months (t = 0.51, *p* = 0.038) (Table 1). With a maximum possible self-care management score of 5, the mean score of the participants was 3.59 ± 0.48, the minimum score was 2.50, and the maximum score was 4.85 (Table 2).

The study variables exhibiting significant correlations with self-care were depression (r = −0.312, *p* < 0.001), knowledge about diabetes (r = 0.299, *p* < 0.001), self-efficacy (r = 0.781, *p* < 0.001), perceived health status (r = 0.280, *p* < 0.001), diabetes distress (r = −0.654, *p* < 0.001), and family support (r = 0.185, *p* < 0.001) (Table 3).

In order to identify factors affecting self-care, multiple linear regression for OLS was used to identify the factors affecting the mean score and QR was used to identify the factors affecting the quantile score. The scores confirmed that no multicollinearity or autocorrelation was present, as the VIF scores for all independent variables used for regression analysis were <10 and the Durbin–Watson score was 1.81.

Based on the OLS analysis results, the factors age, self-efficacy, perceived health status, and diabetes distress explained 71% of the effect on self-care. However, QR results showed that the factors affecting self-care differed in the 10%, 25%, 50%, 75%, and 90% quantiles. In the 10% quantile, the factors were age, smoking within the past 6 months, being educated about diabetes, depression, knowledge related to diabetes, self-efficacy, diabetes distress, and family support. Additional factors identified were as follows: age, smoking within the past 6 months, self-efficacy, and diabetes distress in the 25% quantile; age, self-efficacy, perceived health status, and diabetes distress in the 50% quantile; age, self-efficacy, perceived health status, and diabetes distress in the 75% quantile; and self-efficacy and perceived health status in the 90% quantile (Table 4).

## 4. Discussion

This study was conducted to identify factors associated with various levels of self-care among elderly patients with diabetes using QR. The QR results were compared with those of the OLS method.

In the OLS analysis, factors affecting self-care among elderly diabetic patients were identified as age, self-efficacy, perceived health status, and diabetes distress. Previous studies using OLS found that the factors affecting self-care among elderly or general diabetes patients were self-efficacy (which was also significant in our study), as well as social support, education level, and duration of diabetes (which our study did not identify as significant factors) [5]. The differences between our study results and those of previous studies may be related to the living areas of patients and their differing races and ethnicities.

In the QR analysis, the factors affecting self-care in each quantile differed except for self-efficacy, which was a factor in all quantiles. As a factor affecting self-care levels ranging from low to high, self-efficacy, or the perceived power to take action, has been identified as an important determinant of health-promoting behavior [14]. Previous studies reported that self-efficacy was a strong predictor of self-care and affected metabolic control by increasing the perceived ability to engage in self-care [4]. According to Bandura [14], individuals’ self-efficacy, which he describes as the capability to cope with stressors in one’s life, activates biological systems that mediate the relationship between health and disease. Therefore, to improve self-care activity, promoting self-efficacy is particularly important and should be prioritized in interventions intended to enhance self-care behavior in elderly diabetic patients. 

Age and diabetes distress were identified as factors affecting self-care in the 10%, 25%, 50%, and 75% quantiles, but not the 90% quantile. Age is an individual factor that may affect patients’ approach to self-care and influence their perception of health [33], but it is an uncontrollable factor. Therefore, health providers should focus on developing nursing care plans that will decrease diabetes distress in elderly patients with self-care scores in the 10%, 25%, 50%, and 75% quantiles. The negative effect of diabetes distress was stronger as the quantile increased. Diabetes distress has been identified as an important clinical factor that has negative effects on both blood glucose levels and self-care [34]. Nevertheless, in a previous systematic review of nursing intervention studies addressing diabetes distress, the authors reported that only three of the 17 studies targeted diabetes distress in self-care interventions [34]. Therefore, providing interventions or improving self-care among elderly diabetes patients, except those with a high level of self-care (in the 90% quantile), should include strategies for decreasing diabetes distress.

Among other factors affecting self-care, perceived health status was present in the 50%, 75%, and 90% quantiles; smoking within the past 6 months was present in the 10% and 25% quantiles; and education level, being educated about diabetes, depression, knowledge related to diabetes, and family support were present only in the 10% quantile. What is notable here is that self-care of elderly patients with diabetes in the 10% quantile was affected by many factors compared with the levels of self-care in other quantiles. Our QR results could not be compared with those of other studies because no previous study has identified factors affecting the lowest level of self-care. However, based on our results, health service providers, including nurses in medical clinics, should provide suitable diabetes education and apply interventions to decrease depression and improve family support for elderly patients with diabetes with very low self-care levels, such as those in the 10% quantile. 

Previous studies [35,36,37] developed or adopted self-care improvement programs for elderly people with diabetes. However, in those studies, the same self-care behavior development program was used for both participants who were capable of self-care behavior and those who were not. The studies did not consider the fact that different elements should be taken into consideration depending on the self-care abilities of the subjects involved. In this regard, the present study applied the QR model to clarify the need to consider these different factors according to the self-care behavior level. This study’s results suggest that to improve levels of self-care when developing nursing strategies or health policies, different approaches need to be planned depending on the needs of individual subjects. Hence, health and medical treatment policy makers or clinical nurses need to provide intensive management for subgroups incapable of self-care behavior with different strategies for education, smoking cessation, depression, stress management, family support, etc., rather than planning uniform programs that neglect differences among diabetes patients. Particularly in Korean culture, which takes a serious view of attachment among family members, the reduction of family support may lead to a vicious cycle of increased depression and stress, as well as decreased self-care behavior among elderly patients with diabetes who exhibit inferior self-care abilities. Considering that the subjects of this study were Korean, future studies need to compare factors related to the self-care abilities of subjects in different cultures and examine the results. For groups capable of self-care, simplified programs that include self-efficacy improvement or stress control strategies need to be developed. 

As mentioned above, the QR analysis results differed from those of the OLS analysis. In previous studies [5], analyses using most regression models such as OLS provided only point estimates of the center of the conditional distribution of self-care scores. However, a more complete estimate of the conditional distribution can be obtained in the form of quantiles of self-care scores [19]. For this reason, the use of QR analysis is expected to expand in research studying individuals with multiple health-related characteristics. A limitation of this study is that participants were from the middle-eastern region of Korea, where they visited one public health center and two medical clinics, so caution is needed in generalizing our study results. Further studies will need to consider a longitudinal design to test the causal effects of individual and holistic environmental predictors or to compare factors affecting self-care behavior among elderly patients with type 2 diabetes in culturally different regions. Another limitation was that 65.6% of the participants in the study were early elderly and 34.4% were later elderly, which is similar to the ratio of the elderly population in South Korea but it was too low to represent the entire elderly population. There should be comparative studies done on early and later elderly populations. 

## 5. Conclusions

This study investigated factors affecting self-care scores ranging from low to high among elderly patients with diabetes. Factors affecting self-care scores in previous studies using the OLS model were identified as affecting the mean level of self-care, but those studies did not investigate the factors affecting lower or higher self-care scores of elderly patients with diabetes. In contrast, using QR, this study shows that the self-care of elderly diabetes patients with low self-care scores was affected by many factors compared with that of patients with high self-care scores. These results indicate the importance of clinical staff assessing factors according to the level of self-care behavior when examining patients’ health behaviors and planning intervention strategies.

## Figures and Tables

**Table 1:** Self-care scores with respect to general characteristics of elderly patients with diabetes (*N* = 198).

General Characteristics	*N* (%)	Mean (SD)	t or F	*p*
Gender			1.93	0.055
Male	118 (59.6)	3.65 (0.49)		
Female	80 (40.4)	3.52 (0.47)		
Age (years)			7.76	<0.001
65–69	67 (33.8)	3.80 (0.44)		
70–74	63 (31.8)	3.53 (0.52)		
75–79	41 (20.7)	3.53 (0.41)		
>85	27 (13.6)	3.34 (0.44)		
Education level			3.76	0.012
None	43 (21.7)	3.40 (0.51)		
Primary	129 (65.2)	3.64 (0.45)		
Middle	15 (7.6)	3.59 (0.50)		
High	11 (5.6)	3.85 (0.57)		
Economic status			3.21	0.003
Low	23 (11.5)	3.33 (0.49)		
Middle	170 (86.5)	3.62 (0.47)		
High	5 (2.5)	4.23 (0.18)		
Marital status			−0.51	0.606
Married	187 (94.4)	3.59 (0.49)		
Single	11 (5.6)	3.67 (0.46)		
Educational place			0.92	0.648
Hospital	36 (18.2)	3.37 (0.55)		
Public health center	7 (3.5)	3.62 (0.27)		
Mass media	58 (29.3)	3.70 (0.50)		
Smoking within the past six months			0.51	0.648
Yes	5 (2.5)	3.59 (0.49)		
No	193 (97.5)	3.71 (0.26)		
Drinking within the past six months			2.09	0.038
Yes	25 (12.6)	3.57 (0.49)		
No	173 (87.4)	3.78 (0.35)		
BMI (kg/m^2^)			2.04	0.133
<25	48 (24.2)	3.64 (0.52)		
25–30	79 (39.9)	3.65 (0.41)		
>30	71 (35.9)	3.50 (0.52)		

BMI, body mass index; SD, standard deviation; *p* < 0.05.

**Table 2:** Scores of self-care, depression, knowledge related to diabetes, self-efficacy, perceived health status, diabetes distress, and family support among elderly patients with diabetes (*N* = 198).

Variable	Min	Max	Mean	SD
Self-care	2.50	4.85	3.59	0.48
Depression	0.00	15.00	5.63	4.77
Knowledge related to diabetes	3.00	17.00	8.09	2.60
Self-efficacy	14.00	38.00	26.93	4.85
Perceived health status	6.00	13.00	9.55	1.19
Diabetes distress	1.00	4.29	1.86	0.57
Family support	21.00	48.00	40.23	5.36

SD, standard deviation.

**Table 3:** Relationships among affecting factors and self-care (*N* = 198).

	Self-Care	Depression	Knowledge about Diabetes	Self-Efficacy	Perceived Health Status	Diabetes Distress	Family Support
Self-care	1						
Depression	−0.312 ^2^	1					
Knowledge about diabetes	0.299 ^2^	−0.004	1				
Self-efficacy	0.781 ^2^	−0.430 ^2^	0.287 ^2^	1			
Perceived health status	0.280 ^2^	−0.317 ^2^	0.003	0.525 ^2^	1		
Diabetes distress	−0.654 ^2^	0.351 ^2^	−0.303 ^2^	−0.561 ^2^	−0.180 ^1^	1	
Family support	0.185 ^2^	−0.205 ^2^	0.212 ^2^	0.116	−0.007	−0.258 ^2^	1

^1^*p* < 0.05; ^2^ *p* < 0.001.

**Table 4:** Comparison between ordinary least squares (OLS) and quantile regression (QR) results on the factors affecting self-care (*N* = 198).

	OLS ß (SE)			QR Coef (SE)		
Variables		Q 0.10	Q 0.25	Q 0.50	Q 0.75	Q 0.90
Age	−0.013 ^1^	−0.007 ^1^	−0.012 ^1^	−0.013 ^1^	−0.017 ^1^	−0.022
	(0.005)	(0.003)	(0.005)	(0.004)	(0.007)	(0.011)
Education level	−0.028	0.001	−0.049	−0.057	0.002	0.001
	(0.031)	(0.027)	(0.037)	(0.029)	(0.037)	(0.064)
Economic status	0.058	0.062	0.033	0.067	0.007	0.013
	(0.064)	(0.071)	(0.087)	(0.059)	(0.081)	(0.013)
Illness period	0.004	0.002	0.003	0.004	0.003	0.003
	(0.004)	(0.003)	(0.005)	(0.003)	(0.005)	(0.646)
Smoking within the past six months	−0.063	−0.283 ^2^	−0.228 ^1^	−0.151	−0.004	0.011
	(0.127)	(0.059)	(0.101)	(0.114)	(0.096)	(0.137)
Education about diabetes	0.016	−0.095 ^1^	−0.041	−0.044	0.006	0.053
	(0.043)	(0.033)	(0.037)	(0.041)	(0.067)	(0.121)
Depression	0.006	0.023^2^	0.009	0.004	0.011	0.002
	(0.005)	(0.004)	(0.007)	(0.005)	(0.006)	(0.007)
Knowledge about diabetes	−0.005	−0.014 ^1^	−0.021	−0.011	−0.001	−0.025
	(0.009)	(0.006)	(0.011)	(0.008)	(0.011)	(0.017)
Self-efficacy	0.063 ^1^	0.049 ^1^	0.062 ^2^	0.053 ^2^	0.059 ^2^	0.065 ^1^
	(0.007)	(0.007)	(0.009)	(0.006)	(0.009)	(0.022)
Perceived health status	−0.051 ^1^	−0.008	−0.029	−0.043 ^1^	−0.051 ^1^	−0.071 ^1^
	(0.02)	(0.014)	(0.023)	(0.017)	(0.023)	(0.035)
Diabetes distress	−0.246 ^1^	−0.468 ^1^	−0.403 ^2^	−0.419 ^2^	−0.238 ^2^	−0.163
	(0.045)	(0.024)	(0.044)	(0.043)	(0.091)	(0.209)
Family support	0.005	0.015^1^	0.004	0.006	0.002	0.012
	(0.004)	(0.002)	(0.056)	(0.003)	(0.006)	(0.714)
R2	0.71	0.511	0.507	0.533	0.493	0.461

ß, quantile regression coefficients; Coef, coefficient; OLS, ordinary least squares; QR, quantile regression; Q, quantile; SE, standard error. ^1^ *p* < 0.05; ^2^ *p* < 0.001.
